# Comparison of proximal gastrectomy and total gastrectomy in proximal gastric cancer: a meta-analysis of postoperative health condition using the PGSAS-45

**DOI:** 10.1186/s12885-024-13046-3

**Published:** 2024-10-15

**Authors:** Xiangyu Yang, Zhili Zeng, Ziyue Liao, Caiyu Zhu, Hongyang Wang, Haijuan Wu, Shu Cao, Weizheng Liang, Xiushen Li

**Affiliations:** 1 Department of Traditional Chinese Medicine, Jiangxi Maternal and Child Health Hospital, Nanchang, 330006 Jiangxi China; 2 Department of Gastroenterology and Hepatology, The Second Affiliated Hospital of Chongqing Medical University, No.76 Linjiang Road, Yuzhong District, 400010 Chongqing, China; 3 Department of Breast Oncology, The Second Affiliated Hospital of Guangzhou University of Chinese Medicine, Guangzhou, 510120 Guangdong China; 4 Post-Doctoral Research Center, Guangdong Provincial Hospital of Chinese Medicine, Guangzhou, 510120 Guangdong China; 5 College of Traditional Chinese Medicine, Anhui University of Chinese Medicine, Hefei, China; 6 Central Laboratory, The First Affiliated Hospital of Hebei North University, Zhangjiakou, 075000 Hebei China; 7 Jiangxi Provincial Key Laboratory of Female Reproduction Integrated Traditional Chinese and Western Medicine, Jiangxi Maternal and Child Health Hospital, Nanchang, 330006 Jiangxi China; 8 School of Pharmaceutical, Guangzhou University of Chinese Medicine, Guangzhou, Guangdong China

**Keywords:** Proximal gastrectomy, Proximal gastric cancer, Postoperative health condition, PGSAS-45, Meta-analysis

## Abstract

**Purpose:**

Proximal gastrectomy (PG) offers advantages over total gastrectomy (TG) in enhancing the postoperative nutritional status of patients with proximal gastric cancer (PGC), yet its effect on long-term quality of life is still debated. This study aims to thoroughly compare postoperative health condition outcomes between PG and TG.

**Methods:**

We conducted a systematic search of English-language articles from the PubMed, Web of Science, and Cochrane Library databases, covering studies published up to February 2023. Key evaluation endpoints included surgical outcomes and postoperative health condition, assessed using the Post-Gastrectomy Syndrome Assessment Scale-45 (PGSAS-45).

**Results:**

Six retrospective cohort studies were included in the analysis. The PG group demonstrated no significant negative impact on surgical outcomes compared to the TG group. Notably, patients who underwent PG experienced a superior postoperative health condition, characterized by fewer gastroesophageal reflux symptoms (WMD = -0.106, 95% CI -0.183 to -0.029, *P* < 0.01), less weight loss (WMD = 4.440, 95% CI 3.900 to 4.979, *P* < 0.01), and reduced dietary dissatisfaction (WMD = -0.205, 95% CI -0.385 to -0.025, *P* = 0.03).

**Conclusion:**

This study provides compelling evidence that PG is superior to TG in enhancing postoperative health condition for patients with proximal gastric cancer, without compromising surgical outcomes. However, further rigorous randomized controlled trials are necessary to inform surgical decision-making more effectively.

**Supplementary Information:**

The online version contains supplementary material available at 10.1186/s12885-024-13046-3.

## Introduction

Gastric cancer is the third leading cause of cancer-related deaths, with over 10,000 new diagnoses globally each year. Recently, the incidence of proximal gastric cancer (PGC) has increased worldwide [1], even as the overall incidence of gastric cancer declines in some regions [2]. PGC, located primarily in the upper stomach, presents significant challenges due to its proximity to the gastroesophageal junction. Choosing the appropriate surgical approach involves careful consideration of factors such as tumor location, extent, and patient-specific variables. Nevertheless, the optimal treatment strategy is still debated.

Total gastrectomy (TG), which entails complete removal of the stomach, has been the standard surgical approach for proximal gastric cancer. This method ensures thorough tumor removal and adequate lymph node dissection. However, it often leads to changes in digestive physiology, resulting in postoperative complications such as malabsorption, dumping syndrome, and nutritional deficiencies [3].

Proximal gastrectomy (PG) is an alternative surgical procedure that preserves more than half of the distal stomach [4]. While PG is advantageous for organ and function preservation, it is associated with more severe reflux esophagitis and anastomotic stricture compared to TG [5]. Recent studies indicate that alternative procedures such as jejunal interposition (JI), jejunal pouch interposition (JPI), and double-tract reconstruction (DTR) can significantly reduce these complications and enhance postoperative nutritional outcomes [6].

Long-term survival rates following radical gastrectomy have improved due to enhanced early detection and surgical techniques. Consequently, surgeons’ focus has shifted from merely achieving surgical success to considering the long-term postoperative health condition of patients, including postoperative symptoms, life status, and quality of life (QOL). However, this area remains controversial, as measuring subjective and physical symptoms poses challenges, leading to a lack of uniform evaluation criteria [7]. Previous generic QOL questionnaires, such as the QLQ-30, provide primary quantitative assessments but lack gastrointestinal specificity [8].

This meta-analysis employs the Post-Gastrectomy Syndrome Assessment Scale-45 (PGSAS-45) to systematically review and analyze the postoperative health condition of patients with proximal gastric cancer who underwent TG and PG. It evaluates postoperative symptoms, life status, and QOL while comparing the surgical outcomes of both procedures. The findings from this study may help clinicians choose the most suitable surgical approach for proximal gastric cancer, taking into account both oncological outcomes and postoperative health condition.

## Methods

### Search strategy and data sources

This meta-analysis adhered to the Preferred Reporting Items for Systematic Reviews and Meta-Analyses (PRISMA) guidelines [9]. A thorough literature search was performed across PubMed, Web of Science, and Cochrane Library databases, covering all publications from their inception to February 2023. The search utilized Medical Subject Headings (MeSH) terms such as “stomach neoplasms,” “gastrectomy,” and “quality of life,” along with relevant keywords in titles and abstracts.

### Inclusion and exclusion criteria

Studies were included if they met the following criteria: (1) comparison of PG and TG; (2) presentation of tumor stage or depth of invasion for PGC; and (3) inclusion of surgical outcomes or quality of life data based on PGSAS-45 statistics. In cases where two studies utilized the same cohort, the study with more comprehensive outcomes was chosen.

Studies were excluded based on the following criteria: (1) absence of necessary statistics, such as variance; (2) non-English publications; (3) posters, review articles, commentaries, and abstract-only papers; (4) lack of data on tumor stage and depth of invasion; (5) inclusion of heterogeneous surgical types; (6) inability to convert quality of life data because the PGSAS-45 was not used or did not provide essential outcome measures. The study by Tanizawa, which compared the effects of TG and PG on postoperative health condition using PGSAS-45, was excluded as it focused solely on dumping syndrome indicators without presenting data on other relevant outcomes [10].

### Data extraction and bias assessment

Data extraction for the included studies was conducted independently by two authors (X.Y.Y and Z.L.Z). The extracted data encompassed: (1) study background (authors, year of publication, study design, nationality, and cohort size); (2) cohort characteristics (age, sex, BMI, type of surgery, anastomosis type, and tumor stage); (3) symptoms (esophageal reflux, abdominal pain, meal-related distress, indigestion, diarrhea, constipation, dumping, and total symptom score); (4) life status (changes in body weight, amount of food ingested per meal, need for additional meals, quality of ingestion, and ability for working); (5) QOL (dissatisfaction with symptoms, dissatisfaction with the meal, dissatisfaction at work, dissatisfaction for daily life, physical component summary [PCS], mental component summary [MCS]).

The PGSAS-45 is a detailed questionnaire designed to assess post-gastrectomy syndrome [11, 12]. It comprises 45 questions, including 8 items from the Short Form Health Survey (SF-8) [13], 15 items from the Gastrointestinal Symptom Scale [14, 15], and 22 items deemed clinically important and newly selected by gastric surgeons [14, 15] (Supplementary Table S1).

Additionally, the 23 items related to postoperative symptoms were categorized into 7 subscales: esophageal reflux, abdominal pain, meal-related distress, indigestion, diarrhea, constipation, and dumping. The 19 primary outcome measures were then consolidated and organized into 3 domains: symptoms, life status, and QOL (Supplementary Table S2).

The quality of the included studies was assessed using the Newcastle-Ottawa Quality Assessment Scale (NOS), which has a scoring range from 0 (worst) to 9 (best) [16]. The NOS evaluation focused on three quality parameters: selection of study populations, comparability between groups, and outcome measures. Studies scoring 6 or higher were deemed high quality, while those below 6 were classified as low quality (Supplementary Table S3). Funnel plots were utilized to evaluate publication bias, and no bias was detected (Supplementary Figure S1).

### Statistical analysis

Analyses were conducted using R language version 4.2.1. Dichotomous variables were represented as odds ratios (ORs), while continuous variables were presented as weighted mean differences (WMDs). All results included 95% confidence intervals (CIs) calculated using the Mantel-Haenszel method [17]. Given the high heterogeneity among the studies, a random-effects model was employed; otherwise, a fixed-effects model was applied [18]. When only median and interquartile range data were available, estimates were derived from the mean and standard deviation [19]. Means and standard deviations were calculated based on the median, interquartile range, and study sizes. All tests were two-sided, with *P* < 0.05 indicating statistical significance. Cochran Q and *I*² statistics assessed between-study heterogeneity [20]. Sensitivity analyses involved removing individual studies to evaluate their impact on overall outcomes and identify sources of significant heterogeneity.

## Results

### Study characteristics

**Fig. 1:**
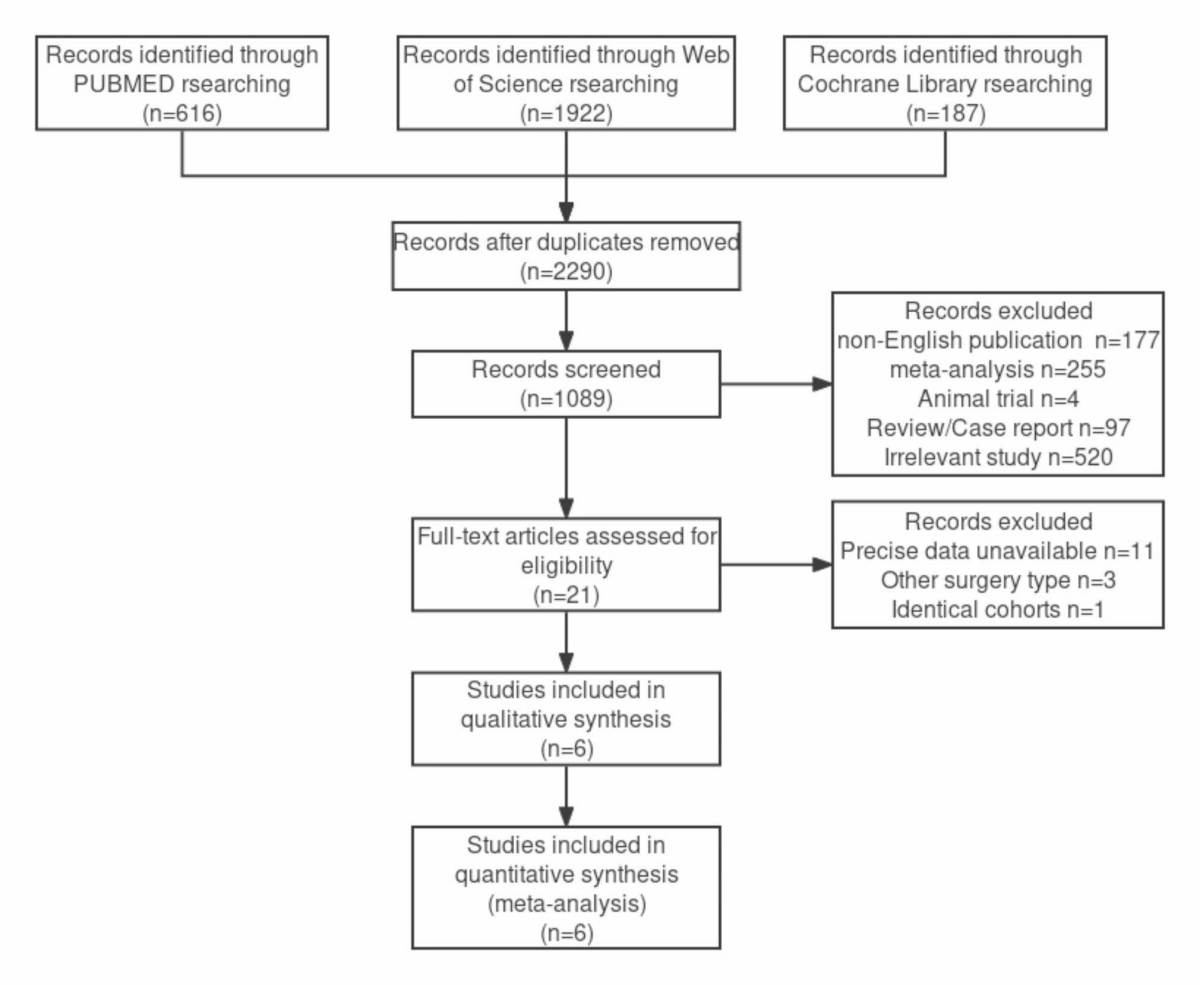
Flow diagram for the meta-analysis

A total of 2,290 potential studies were identified across PubMed, Web of Science, and Cochrane Library databases, resulting in 6 eligible studies without duplication [7, 21–25]. These studies included 2,929 patients, with 904 undergoing PG and 1,604 receiving TG (Fig. 1; Table 1). Five studies were conducted in Japan, and one in Korea. Surgical approaches varied between open and laparoscopic methods, with three studies using laparoscopic techniques. Three studies did not impose restrictions on the gastrointestinal tract (GI), while the others employed various reconstruction methods, including esophagogastrostomy (EG), JI, JPI and DTR. Notably, Lee, SW analyzed the postoperative health condition of PG patients separately for EG and DTR reconstruction subgroups. Table 1 summarizes the characteristics of the included studies, with sample size, age, gender, and tumor stage presented as mean ± standard deviation or median (interquartile range).

**Table 1:** Characteristics of studies included in the meta-analysis

Study publish year	Study design	Country	Patients criteria	Surgery type	Approach (*N*)	Reconstruction	Postoperative time	Gender (male)	Age	NOS
Kunisaki, C. 2022	retrospective	Japan	I-IV	Open/Laparoscopy	PG (518) TG (1020)	EG/DTR/JI/JPI RY/DTE/JI	> 6 months	394 743	69.8 ± 9.5 68.3 ± 10.4	7
Lee, SW. 2021	retrospective	Japan	I-IV	Open/Laparoscopy	PG (120) TG (86)	EG/DTR/JI/JPI RY	> 6 months	103 75	67.9 ± 10.1 67.4 ± 10.1	8
Nishigori, T. 2016	retrospective	Japan	I	Laparoscopy	PG (20) TG (42)	EG RY	> 1 year	15 28	66.2 ± 13.4 64.4 ± 12.2	9
Takiguchi, N. 2013	retrospective	Japan	I	Open/Laparoscopy	PG (193) TG (393)	EG/JI/JPI RY	> 1 year	139 276	63.7 ± 7.7 63.4 ± 9.2	8
Tsumura, T. 2020	retrospective	Japan	I-II	Laparoscopy	PG (19) TG (17)	DFT RY	3 years (median time)	16 11	71.2 ± 8.0 68.9 ± 12.1	9
Park, J.Y. 2018	retrospective	Korea	I-III	Laparoscopy	PG (34) TG (46)	DTR RY	6 months to 3 years	26 22	64.1 ± 12.256.7 ± 11.8	9

PG, proximal gastrectomy; TG, total gastrectomy; EG, esophagogastrostomy; DTR, double-tract reconstruction; JI, jejunal interposition; JPI, jejunal-pouch interposition

### Patient’s clinicopathologic features

Analysis of patient characteristics revealed a significant age difference (WMD = 1.061, 95% CI 0.269 to 1.852, *P* < 0.01). No significant differences were found in gender (OR = 1.039, 95% CI 0.990 to 1.090, *P* = 0.12) or preoperative BMI (WMD = -0.031, 95% CI -0.296 to 0.233, *P* = 0.82) (Table 2). There was no notable difference in D1+ lymph node dissection between patients in the PG and TG groups (OR = 1.315, 95% CI 0.812 to 2.130, *P* = 0.27). The TG group had a higher proportion of combined cholecystectomies compared to the PG group (OR = 0.411, 95% CI 0.318 to 0.531, *P* < 0.01) (Table 2).

**Table 2:** Subgroup meta-analysis of comparison between PG and TG

Subgroup	No. of studies	OR/WMD	95% CI	*p* value	Heterogeneity (I^2^)	Effect model
Basic characteristics						
Age	6	1.061	0.269, 1.852	< 0.01	0%	Fixed
Gender (male)	6	1.039	0.990, 1.090	0.12	0%	Fixed
BMI (kg/m^2^)	6	-0.031	-0.296, 0.233	0.82	0%	Fixed
Stage I	6	1.472	1.414, 1.533	< 0.01	100%	Random
D1 + lymphnode dissection	4	1.315	0.812, 2.130	0.27	99%	Random
Combined cholecystectomy	4	0.411	0.318, 0.531	< 0.01	0%	Fixed
Surgical outcomes						
Operation time(minutes)	3	-34.719	-44.396, -25.042	< 0.01	0%	Fixed
Intraoperative blood loss (ml)	3	-36.51	-172.72, 99.69	0.19	95%	Random
Postoperative complications	3	0.656	0.369, 1.167	0.15	0%	Fixed
Postoperative symptoms						
Esophageal reflux SS	5	-0.106	-0.183, -0.029	< 0.01	43%	Fixed
Abdominal pain SS	5	0.076	-0.162, 0.314	0.53	93%	Random
Meal-related distress SS	5	-0.185	-0.482, 0.112	0.22	89%	Random
Indigestion SS	5	-0.134	-0.292, 0.023	0.09	74%	Random
Diarrhea SS	5	-0.360	-0.640, -0.079	0.01	88%	Random
Constipation SS	5	0.073	-0.129, 0.274	0.48	73%	Random
Dumping SS	5	-0.433	-0.773, -0.093	0.01	90%	Random
Total symptom score	5	-0.056	-0.117, 0.006	0.08	39%	Fixed
Postoperative living status						
Change in BW (%)	5	4.440	3.900, 4.979	< 0.01	95%	Random
Ingested amount of food per meal	5	0.143	-0.005, 0.290	0.06	5%	Fixed
Necessity for additional meals	5	-0.240	-0.383, -0.096	0.01	71%	Random
Quality of ingestion SS	5	-0.130	-0.319, 0.059	0.18	74%	Random
Ability for working	5	0.125	-0.096, 0.346	0.27	83%	Random
Postoperative QOL						
Dissatisfaction with symptoms	5	-0.225	-0.649, 0.199	0.30	97%	Random
Dissatisfaction with the meal	5	-0.205	-0.385, -0.025	0.03	68%	Random
Dissatisfaction at working	5	-0.032	-0.293, 0.230	0.81	83%	Random
Dissatisfaction for daily life SS	5	-0.162	-0.422, 0.099	0.22	90%	Random
Physical component summary	5	-0.718	-2.099, 0.664	0.31	85%	Random
Mental component summary	5	-0.751	-1.798, 0.296	0.16	71%	Random

PG, proximal gastrectomy; TG, total gastrectomy; OR, odds ratio; WMD, weighted mean difference; CI, confidence interval; SS, subscale; BW, body weight; QOL, quality of life

### Surgical outcomes

Three studies assessed surgical outcomes. The PG group had a shorter operative time compared to the TG group (WMD = -34.719, 95% CI -44.396 to -25.042, *P* < 0.01). No significant differences were observed in other outcomes, including intraoperative bleeding (WMD = -36.51, 95% CI -172.72 to 99.69, *P* = 0.19) and postoperative complications (OR = 0.656, 95% CI 0.369 to 1.167, *P* = 0.15) (Table 2).

### Postoperative symptoms

**Fig. 2:**
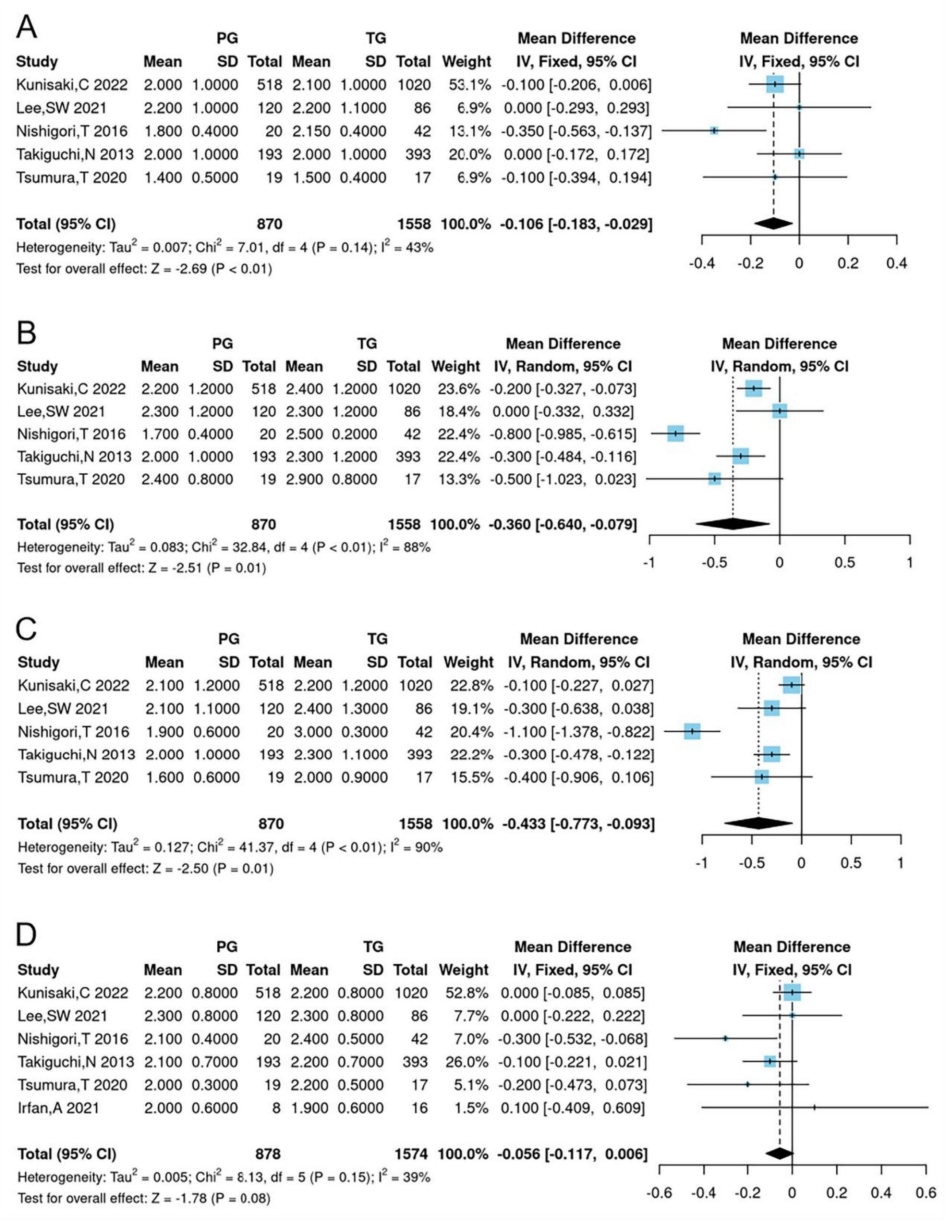
Forest plots for comparing postoperative symptoms between proximal gastrectomy and total gastrectomy. (**A**) esophageal reflux subscale, (**B**) diarrhea subscale, (**C**) dumping subscale, (**D**) total symptom score

The analyses of postoperative symptoms included esophageal reflux, abdominal pain, meal-related distress, indigestion, diarrhea, constipation, dumping and total symptom score. As shown in Table 2; Fig. 2, no significant difference was found in the total symptom score between the PG and TG groups (WMD = -0.056, 95% CI -0.117 to -0.006, *P* = 0.08). However, the PG group reported significantly lower scores for esophageal reflux (WMD = -0.106, 95% CI -0.183 to -0.029, *P* < 0.01), diarrhea (WMD = -0.360, 95% CI -0.640 to -0.079, *P* = 0.01) and dumping (WMD = -0.433, 95% CI -0.773 to -0.093, *P* = 0.01) compared to the TG group. Other postoperative symptom scores showed no significant differences between the groups (Table 2; Fig. 2).

### Postoperative living status

**Fig. 3:**
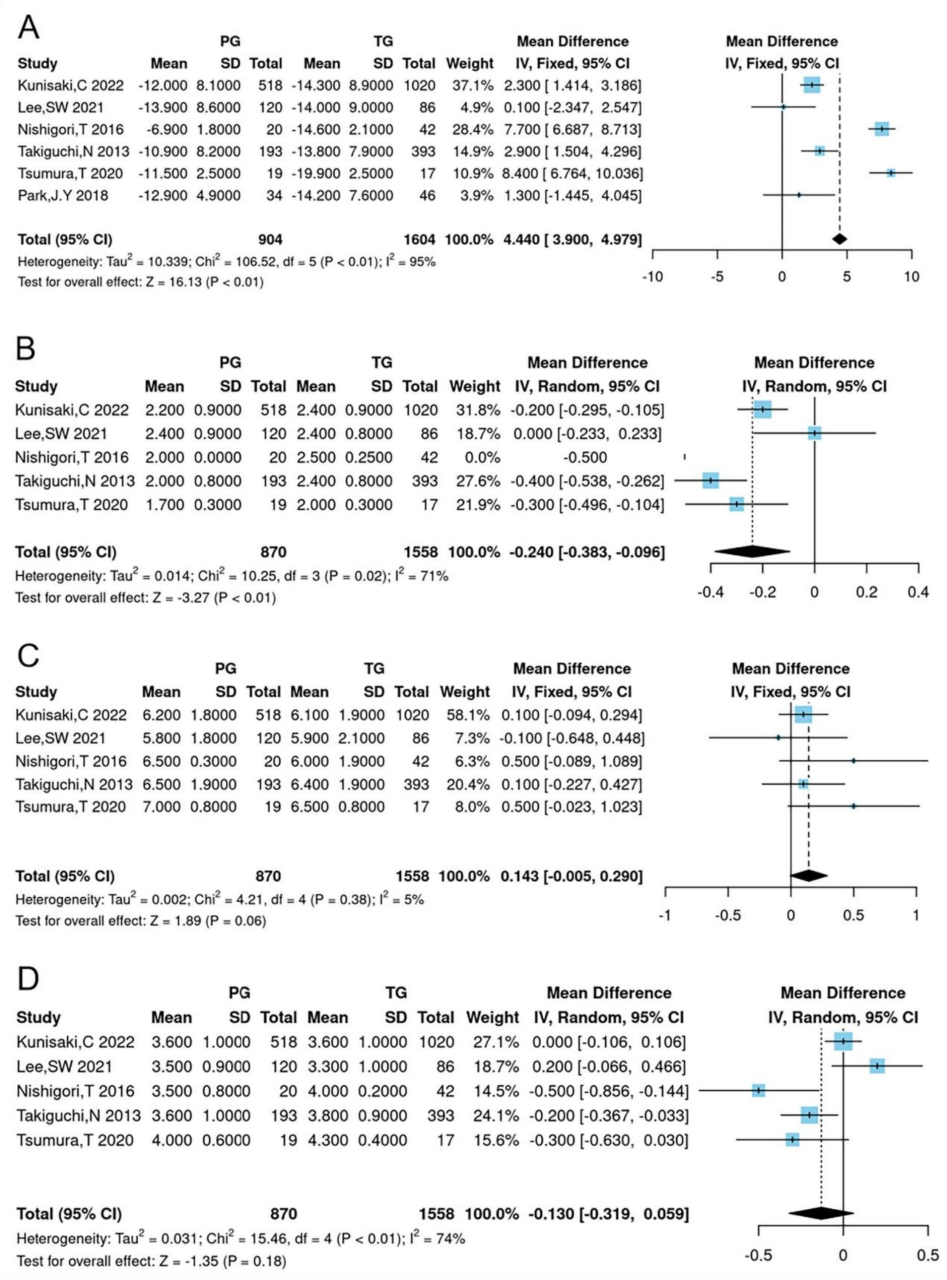
Forest plots for comparing postoperative living status between proximal gastrectomy and total gastrectomy. (**A**) change in BW (%), (**B**) the necessity for additional meals, (**C**) ingested amount of food per meal, (**D**) quality of ingestion

The meta-analysis of operative outcomes assessed several factors, including change in body weight, ingested amount of food consumed per meal, necessity for additional meals, quality of ingestion, and ability for working. Results indicated that the change in body weight was significantly less in the PG group compared to the TG group (WMD = 4.440, 95% CI 3.900 to 4.979, *P* < 0.01) (Table 2; Fig. 3).

Furthermore, the PG group demonstrated a significantly lower need for additional meals than the TG group (WMD = -0.240, 95% CI -0.383 to -0.096, *P* = 0.01), indicating a potential improvement in postoperative nutrition for PG patients. However, the ingested amount of food per meal (WMD = 0.143, 95% CI -0.005 to 0.290, *P* = 0.06), quality of ingestion (WMD = -0.130, 95% CI -0.319 to 0.059, *P* = 0.18), and the ability for working (WMD = 0.125, 95% CI -0.096 to 0.346, *P* = 0.27) did not show significant differences between the two groups (Table 2; Fig. 3).

### Postoperative QOL

**Fig. 4:**
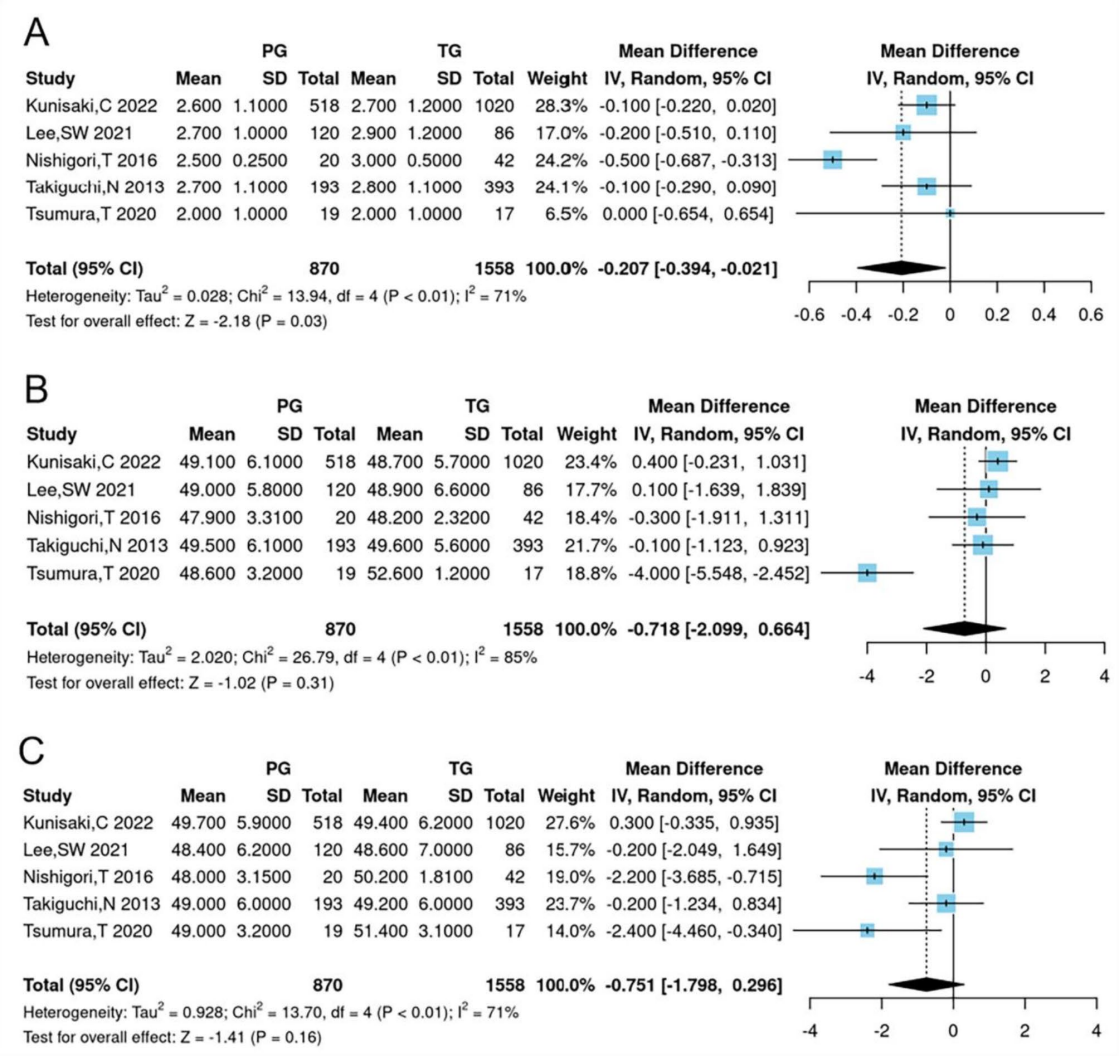
Forest plots for comparing postoperative QOL between proximal gastrectomy and total gastrectomy. (**A**) dissatisfaction with the meal, (**B**) physical component summary of SF-8, (**C**) mental component summary of SF-8

The analysis of postoperative quality of life (QOL) focused on six areas: dissatisfaction with symptoms, dissatisfaction at the meal, dissatisfaction at working, dissatisfaction for daily life, and the physical and mental component summaries of the SF-8. Patients who underwent PG were less likely to experience dissatisfaction at the meal compared to those who had TG (WMD = -0.205, 95% CI -0.385 to -0.025, *P* = 0.03), supporting the notion of improved nutritional status with PG (Table 2; Fig. 4).

Dissatisfaction in other areas was comparable between the two groups, as detailed in Table 2. Besides, there were no differences in the ph ysical component summary of SF-8 (WMD = -0.717, 95% CI -2.098 to 0.663, *P* = 0.31) or the mental component summary (WMD = -0.751, 95% CI -1.798 to 0.296, *P* = 0.16) between the two groups (Table 2; Fig. 4).

## Discussion

This study summarizes the current evidence comparing PG and TG as treatment options for PGC. We utilized the PGSAS-45 scale to assess postoperative health condition and compare surgical outcomes. Given the absence of prospective or randomized controlled studies on this topic, our analysis included only retrospective studies, which may introduce various biases. Nonetheless, this is the first meta-analysis to employ the PGSAS-45 to evaluate postoperative health condition in gastric cancer patients who underwent PG and TG, providing new insights for clinicians in selecting appropriate surgical approaches.

Research on the postoperative nutritional status following PG and TG has shown the advantages of PG [26]. However, the absence of standardized criteria for assessing postoperative health condition underscores the need for further exploration in this area. Long-term health condition post-gastrectomy is increasingly recognized as essential. Previous studies relied on generic quality of life questionnaires, such as the QLQ-30, which lack gastrectomy-specific items and may not fully capture the nuances of postoperative health condition [23, 27]. Our study employed the PGSAS-45, validated for gastric cancer patients, which includes specific items relevant to gastrectomy [10].

Our findings indicate that patients who underwent PG experience fewer symptoms of diarrhea and dumping syndrome compared to those undergoing TG, consistent with prior research [28, 29]. The mechanisms behind this observation may relate to the preservation of the pyloric sphincter and stomach’s reservoir function, which could slow food passage through the digestive tract.

Additionally, the extent of gastroesophageal reflux disease (GERD) in PG group was less severe to that in TG group. Interestingly, prior studies reported a high incidence of postoperative anastomotic strictures and reflux symptoms in PG patients, attributed to the disruption of anti-reflux structures and vagus nerve severance during surgery [30, 31]. The discrepancies in findings may reflect advancements in PG anti-reflux techniques. Various measures, including DTR, JI, JPI, and the double-flap technique (DFT), have been developed to enhance reflux outcomes while maintaining the benefits of PG [32, 33]. Our analysis included studies utilizing different improved anastomosis techniques, such as DFT, JI, JPI, and DTR. We excluded the study by Nishigori et al. due to its laparoscopic hand-sewn technique affecting subjective reflux assessments, and subsequent analysis showed similar reflux symptoms (WMD = -0.069, 95% CI -0.152 to 0.014, *P* = 0.10). This suggests that PG-EG with effective anti-reflux measures may not adversely affect reflux symptoms. However, given the limited studies and ongoing debates about gastrointestinal reconstruction, further research is necessary to clarify these results.

Our analysis indicated that patients undergoing PG experienced less weight loss than those undergoing TG, leading to a better nutritional profile. This aligns with previous studies [28, 29, 34, 35]. One hypothesis is that proximal gastrectomy preserves ghrelin-producing cells in the stomach, which stimulate appetite and promote food intake; this remaining ghrelin may help maintain appetite and reduce the risk of weight loss.

While preserving distal gastric function is thought to result in better QOL, our analysis found almost no significant differences in QOL section between the PG and TG groups, particularly in the physical and psychological components. The only notable difference was the reduced need for additional meals in the PG group, further supporting the improved nutritional profile associated with PG. We speculate that the preservation of the stomach’s reservoir function and pyloric sphincter in proximal gastrectomy may help regulate food intake and maintain a sense of fullness.

Nevertheless, PG is a recommended surgical option for gastric cancer patients due to its lower invasiveness, reduced postoperative anemia, and better vitamin B12 levels [26, 36]. Additionally, DTR is preferentially recommended for gastrointestinal reconstruction after PG, as it offers superior anti-reflux effects while preserving some digestive and storage functions, enhancing nutrient absorption, including vitamin B12 [23, 37].

We also examined the impact of surgical approach on treatment outcomes in PGC. Our analysis revealed that the PG and TG groups had comparable perioperative outcomes, with no significant differences in intraoperative blood loss or postoperative complications. However, the PG group had a shorter operative time, likely due to its simpler surgical steps. It is worth noting that Tsumura’s study reported paradoxically more bleeding in the PG group, which was attributed to misclassifying gastric fluid spillage as blood loss. Consequently, we excluded this study from the analysis of intraoperative blood loss [25].

While PG improves postoperative health condition compared to TG, the development of remnant gastric cancer (RGC) after PG remains a critical concern, significantly affecting long-term survival. RGC refers to new cancers arising in the remaining gastric tissue after gastrectomy for benign or malignant conditions, with an overall incidence of around 2.6% [38]. After PG, however, the incidence is notably higher, ranging from 5.0–8.9% [4, 39–42]. Risk factors for RGC include smoking, Helicobacter pylori infection, and atrophic gastritis [39, 43]. To ensure early detection and treatment, annual gastroscopy is recommended for at least five years following surgery [44]. Some researchers advocate extending this follow-up to 20 years to enhance early detection [45]. Special attention should be given during gastroscopy to the pseudo-fundus, which is often obscured by food debris, potentially delaying the diagnosis of RGC at more advanced stages [46]. Therefore, thorough cleaning of this area is crucial to prevent missed diagnoses.

Early-stage RGC, like primary gastric cancer, can often be managed with endoscopic treatments, while advanced cases may require additional surgery [44, 47]. Advances in laparoscopic techniques now allow laparoscopic completion total gastrectomy to be as effective as open surgery, providing a safe and viable option for patients with advanced RGC [48]. Moreover, there is evidence suggesting that patients with RGC may benefit from adjuvant chemotherapy and immunotherapy [49, 50]. However, as most current studies are retrospective, further prospective research is needed to provide clearer guidance on the management and surveillance of RGC following PG.

Despite adhering to strict inclusion and exclusion criteria, several limitations should be acknowledged. The number of included studies was limited, primarily due to varying quality of life assessment scales, which complicated the inclusion of literature with consistent effect sizes. The reliance on retrospective cohort studies, without prospective cohorts or randomized controlled trials, increases the potential for bias, weakening the strength of the evidence. While the funnel plot did not show publication bias, its presence cannot be completely ruled out. Additionally, variations in surgical techniques, particularly the reconstructive methods used after PG (EG, JI, and DTR), may have affected the outcomes, making direct comparisons more difficult. Subgroup analyses on different anastomosis methods should be considered when sufficient studies are available. Lastly, the lack of specific postoperative time settings in the included studies increased variability in outcome measurements, thereby reducing the reliability of our conclusions.

## Conclusion

In conclusion, proximal gastrectomy is a preferred surgical option over total gastrectomy for patients with proximal gastric cancer, as it offers improved postoperative health condition regarding nutrition and dietary factors without significantly compromising surgical outcomes. Future randomized controlled trials should compare reconstruction modalities, including DTR, EG, and JI, to identify the optimal approach for long-term health condition following PG.

## Electronic supplementary material

Below is the link to the electronic supplementary material.

Supplementary Material 1

Supplementary Material 2

Supplementary Material 3

## Data Availability

No datasets were generated or analysed during the current study.

## Abbreviations

PGC: Proximal gastric cancer
TG: Total gastrectomy
PG: Proximal gastrectomy
JI: Jejunal interposition
JPI: Jejunal pouch interposition
DTR: Double-tract reconstruction
PGSAS-45: Post-Gastrectomy Syndrome Assessment Scale-45
QOL: Quality of life
PRISMA: Preferred Reporting Items for Systematic Reviews and Meta-Analyses
MeSH: Medical Subject Headings
PCS: Physical component summary
MCS: Mental component summary
SF-8: 8 items from the Short Form Health Survey
NOS: Newcastle-Ottawa Quality Assessment Scale
WMD: Weighted mean difference
CI: Confidence interval
GI: Gastrointestinal tract
EG: Esophagogastrostomy
GERD: Gastroesophageal reflux disease
DFT: Double-flap technique
RGC: Remnant gastric cancer
