# Supplementary material for: Comparison of proximal gastrectomy and total gastrectomy in proximal gastric cancer: a meta-analysis of postoperative health condition using the PGSAS-45
Source: BMC Cancer. 2024 Oct 15;24:1282. doi: 10.1186/s12885-024-13046-3 (PMC11481723; doi:10.1186/s12885-024-13046-3)
Supplement: Supplementary file 1 — Supplementary Material 1 [file 12885_2024_13046_MOESM1_ESM.docx]

**Supplementary Information**


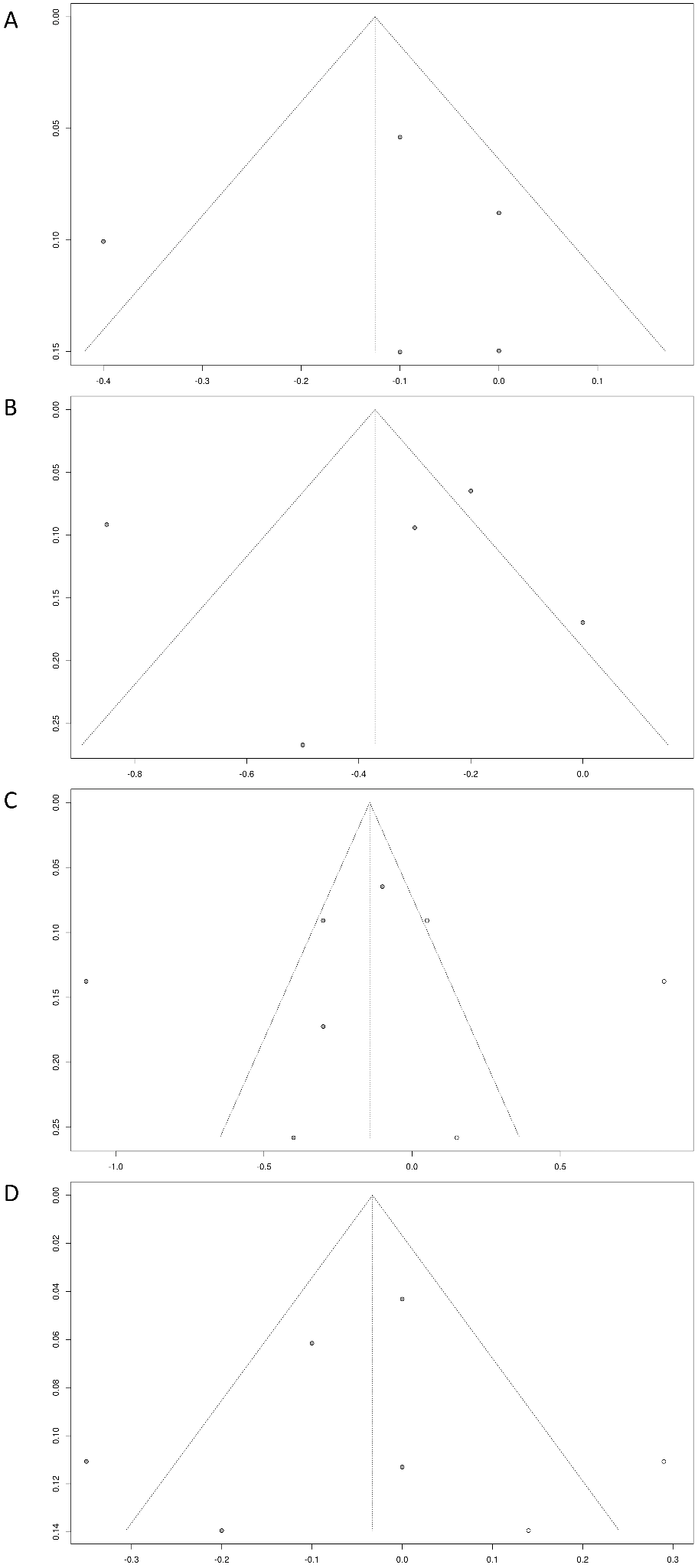


**Supplementary Fig. 1** Funnel plots of publication biases for comparing postoperative symptoms between proximal gastrectomy and total gastrectomy. (A) esophageal reflux subscale, (B) diarrhea subscale, (C) dumping subscale, (D) total symptom score.


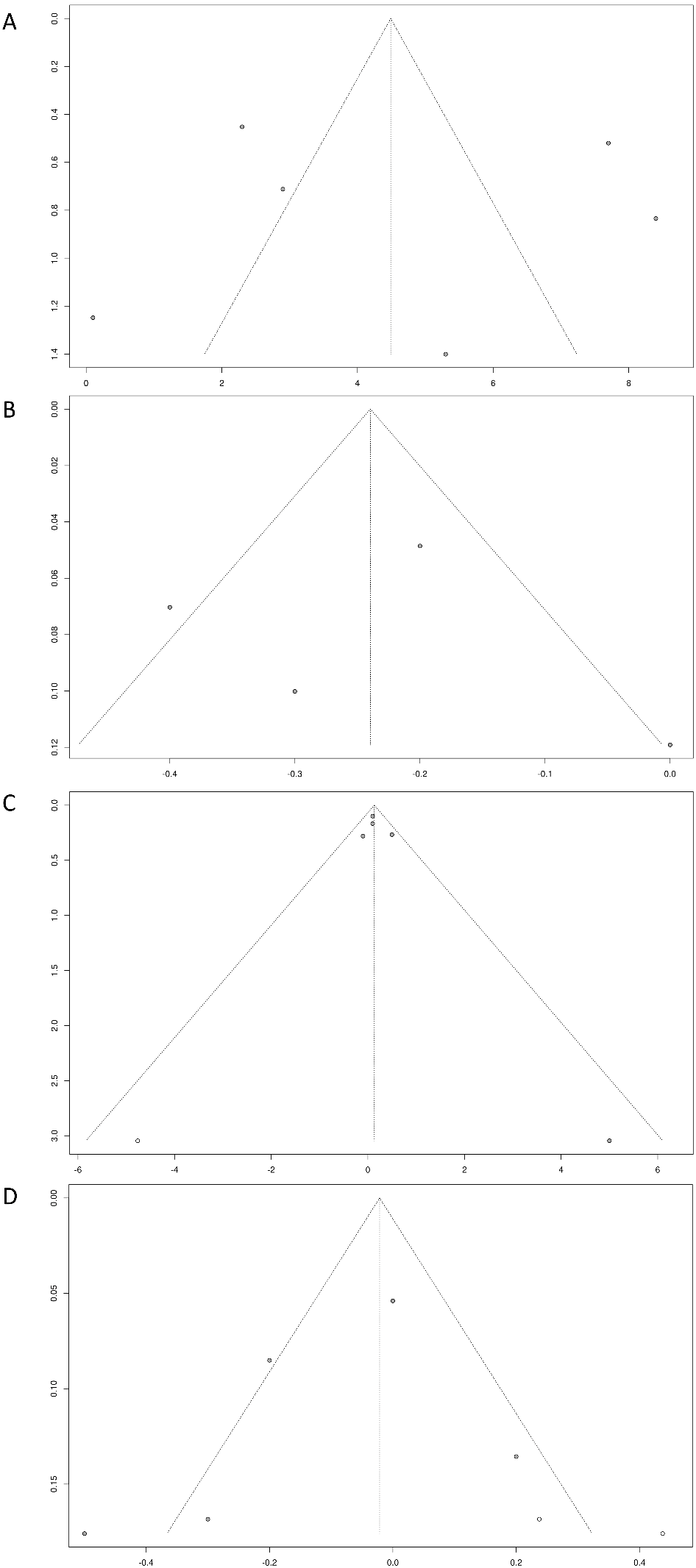


**Supplementary Fig. 2** Funnel plots of publication biases for comparing postoperative living status between proximal gastrectomy and total gastrectomy. (A) change in BW (%), (B) the necessity for additional meals, (C) ingested amount of food per meal, (D) quality of ingestion.


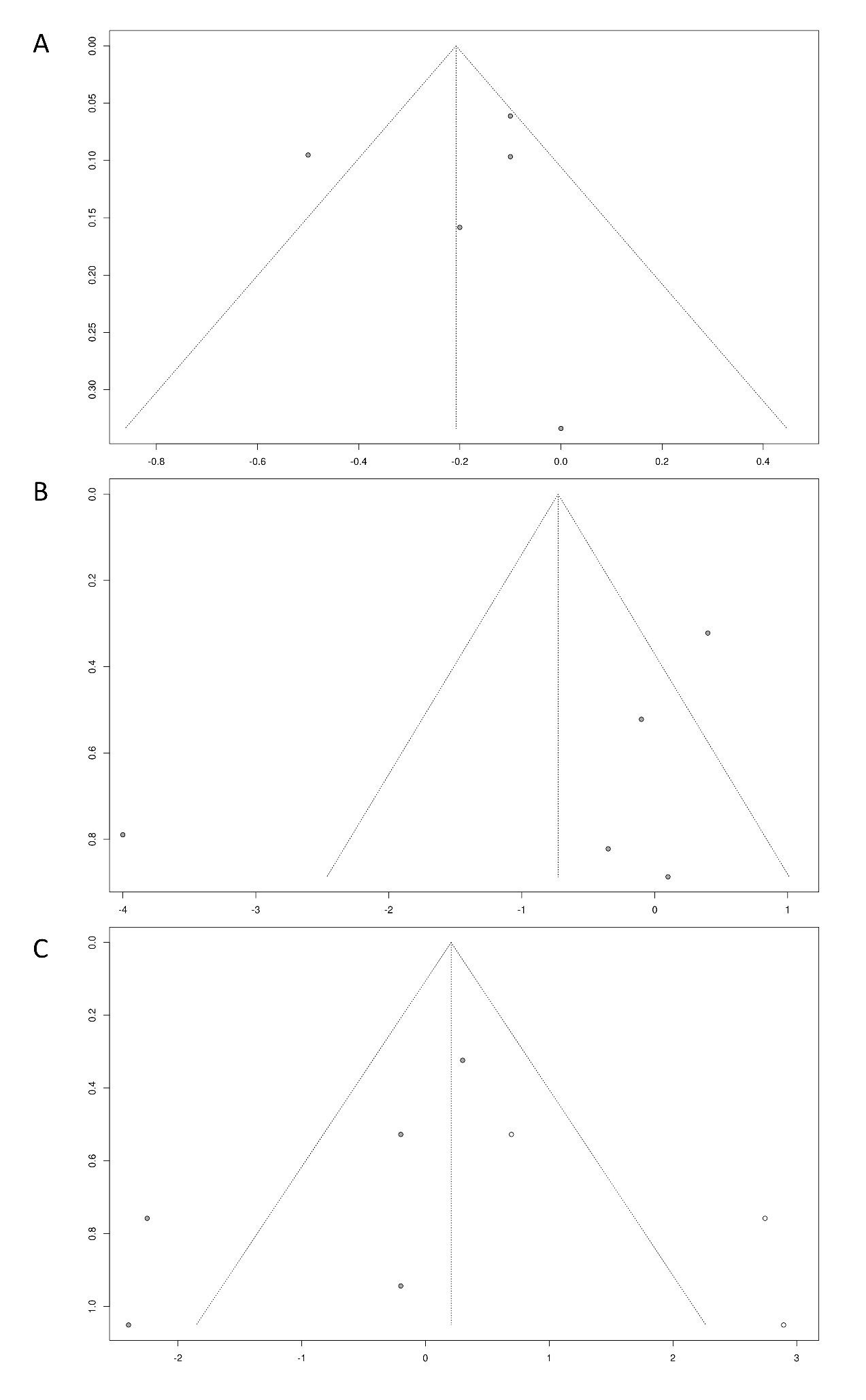


**Supplementary Fig. 3** Funnel plots of publication biases for comparing postoperative QOL between proximal gastrectomy and total gastrectomy. (A) dissatisfaction with the meal, (B) physical component summary of SF-8, (C) mental component summary of SF-8.

**Supplementary Table 1** Structure of the Postgastrectomy Syndrome Assessment Scale-45 (PGSAS-45)

| **Domains** | **Subdomains** | **Items** | **Subscales** |
| --- | --- | --- | --- |
| QOL | SF-8 (QOL) | 1 Physical functioninga  2 Role physicala  3 Bodily paina  4 General healtha  5 Vitalitya  6 Social functioninga  7 Role emotionala  8 Mental healtha | PCSa (items 1–8)  MCSa (items 1–8) |
| Symptoms | GSRS (symptoms)  Symptoms | 9 Abdominal pain  10 Heartburn  11 Acid regurgitation  12 Sucking sensation in the epigastrium  13 Nausea and vomiting  14 Borborygmus  15 Abdominal distension  16 Eructation  17 Increased fatus  18 Decreased passage of stools  19 Increased passage of stools  20 Loose stools  21 Hard stools  22 Urgent need for defecation  23 Feeling of incomplete evacuation  24 Bile regurgitation  25 Sensation of food sticking  26 Postprandial fullness  27 Early satiation  28 Lower abdominal pain  29 Number and type of early dumping symptoms  30 Early dumping general symptoms  31 Early dumping abdominal symptoms  32 Number and type of late dumping symptoms  33 Late dumping symptoms | Esophageal reflux subscale (items 10, 11, 13, 24)  Abdominal pain subscale (items 9, 12, 28)  Meal-related distress subscale (items 25–27)  Indigestion subscale (items 14– 17)  Diarrhea subscale (items 19, 20, 22)  Constipation subscale (items 18, 21, 23)  Dumping subscale (items 30, 31, 33)  Total symptom score (above seven subscales) |
| Living status | Meals (amount)  Meals (quality)  Meals (amount)  Social activity | 34 Ingested amount of food per meal^a^  35 Ingested amount of food per daya  36 Frequency of main meals  37 Frequency of additional meals  38 Appetitea  39 Hunger feelinga  40 Satiety feelinga  41 Necessity for additional meals  42 Ability for working | Quality of ingestion subscalea (items 38–40) |
| QOL | Dissatisfaction (QOL) | 43 Dissatisfaction with symptoms  44 Dissatisfaction at the meal  45 Dissatisfaction at working | Dissatisfaction for daily life subscale (items 43–45) |

*QOL* quality of life, *SF-8* Short-Form Health Survey, *GSRS* Gastrointestinal Symptom Rating Scale, *PCS* physical component summary, *MCS* mental component summary

aFor the indicated items or subscales, a higher score indicated a better condition. For other items or subscales, a higher score indicated a worse condition. Each subscale was calculated as the mean of composed items or subscales, except for the PCS or MCS of SF-8. Items 29 and 32 did not have scores. These items were separately analyzed

**Supplementary Table 2** Domains and main outcome measures

| Domains | Subdomains | Main outcome measures |
| --- | --- | --- |
| Symptoms | Subscales  Total | Seven symptom subscales  Esophageal refux  Abdominal pain  Meal-related distress  Indigestion  Diarrhea  Constipation  Dumping  Total symptom score |
| Living status | Body weight Meals (amount)  Meal (quality)  Work | Change in body weight (%)a Ingested amount of food per meala Necessity for additional meals Quality of ingestion subscalea Ability for working |
| QOL | Dissatisfaction  SF-8 | Dissatisfaction with symptoms Dissatisfaction with the meal Dissatisfaction at working Dissatisfaction for daily life subscale  PCSa  MCSa |

*QOL* quality of life, *SF-8* Short-Form Health Survey, *PCS* physical component summary, *MCS* mental component summary

aFor the indicated items or subscales, a higher score indicated a better condition. For other items or subscales, a higher score indicated a worse condition. Each subscale was calculated as the mean of com- posed items or subscales, except for the PCS or MCS of SF-8.

| **Supplementary Table 3** Quality evaluation of the eligible studies with Newcastle–Ottawa scale. | | | | | | | | | |
| --- | --- | --- | --- | --- | --- | --- | --- | --- | --- |
| Study | Selection | | | | Comparability | | Outcome | | |
|  | Representative-ness | Selection of  non-exposed | Ascertainment  of exposure | Outcome not present at start | Comparability on most important factors | Comparability on other risk factors | Assessment of outcome | Long enough follow-up (median≥1 year) | Adequacy  (completeness) of follow-up |
| Kunisaki et al. | * | * | * | * | - | - | * | * | * |
| Lee et al. | * | * | * | * | * | - | * | * | * |
| Nishigori et al. | * | * | * | * | * | * | * | * | * |
| Takiguchi et al. | * | * | * | * | - | * | * | * | * |
| Park et al. | * | * | * | * | * | - | * | * | * |
| Tsumura et al. | * | * | * | * | * | * | * | * | * |
| *indicates criterion met; - indicates significant of criterion not met. | | | | | | | | | |
