# Supplementary material for: Comparison of proximal gastrectomy and total gastrectomy in proximal gastric cancer: a meta-analysis of postoperative health condition using the PGSAS-45
Source: BMC Cancer. 2024 Oct 15;24:1282. doi: 10.1186/s12885-024-13046-3 (PMC11481723; doi:10.1186/s12885-024-13046-3)
Supplement: Supplementary file 3 — Supplementary Material 3 [file 12885_2024_13046_MOESM3_ESM.docx]

**Identification of studies via databases and registers**

Records removed *before screening*:

Duplicate records removed (n = 435)

Records marked as ineligible by automation tools (n = 0)

Records removed for other reasons (n = 0)

Records identified from*:

Databases (n = 2290)

Registers (n = 0)

**Identification**

Records screened

(n = 1089)

Records excluded**

(n = 1053)

Reports sought for retrieval

(n = 36)

Reports not retrieved

(n = 15)

**Screening**

Reports assessed for eligibility

(n = 21)

Reports excluded:

Precise data unavailable (n = 11)

Use of heterogeneous surgery types (n = 3)

Identical cohorts (n = 1)

Studies included in review

(n = 6)

Reports of included studies

(n = 6)

**Included**

*Consider, if feasible to do so, reporting the number of records identified from each database or register searched (rather than the total number across all databases/registers).

**If automation tools were used, indicate how many records were excluded by a human and how many were excluded by automation tools.

*From:*  Page MJ, McKenzie JE, Bossuyt PM, Boutron I, Hoffmann TC, Mulrow CD, et al. The PRISMA 2020 statement: an updated guideline for reporting systematic reviews. BMJ 2021;372:n71. doi: 10.1136/bmj.n71

For more information, visit: <http://www.prisma-statement.org/>
